# Postoperative anastomotic leakage after colon cancer surgery may reduce long-term survival

**DOI:** 10.1007/s00384-026-05171-1

**Published:** 2026-06-12

**Authors:** S. Müller, P. Koeller, A. Hendricks, R. Seggewiss-Bernhardt, S. Flemming, C. T. Germer, J. F. Lock

**Affiliations:** 1 Department of General, Visceral, Transplant, Vascular and Paediatric Surgery, University Hospital Wuerzburg, 97080 Wuerzburg, Germany; 2 Comprehensive Cancer Centre Mainfranken, University of Wuerzburg, 97080 Wuerzburg, Germany

**Keywords:** Colorectal cancer, Colon cancer surgery, Anastomotic leakage, Overall survival, Postoperative complications

## Abstract

**Background:**

Anastomotic leakage (AL) remains a major cause of postoperative morbidity and mortality after colon cancer surgery and has been discussed potentially impairing long-term survival. However, most available data originate from rectal cancer or mixed cohorts including colon and rectal cancer. Therefore, we investigated the association between AL and overall survival (OS) focusing only on colon cancer.

**Methods:**

We identified all patients who underwent surgery for colon cancer at the University Hospital of Wuerzburg between 1 January 2019 and 31 December 2021 via the institutional tumor registry (Onkostar). Rectal cancer patients were not included in the present study. Clinical, surgical, pathologic, and follow-up data (last follow-up June 30, 2025) were extracted and complemented by hospital information systems. OS was defined as time from diagnosis to death or last contact. Multivariable Cox proportional hazards regression—with candidate variables including age, UICC stage, R-status, emergency presentation, diabetes, and immune status—was conducted using stepwise forward selection (entry *p* < 0.05, exit *p* > 0.10). Hazard ratios (HRs) with 95% confidence intervals (CIs) were reported and Kaplan–Meier curves plotted to visualize group differences.

**Results:**

The cohort comprised 166 patients; 15 (9%) developed AL. The overall mean survival was 59.6 months (95% CI 55.6–63.7). Baseline tumor localization, UICC stage, and R0 resection rates (98%) were similar between patients with and without AL. Rectal anastomoses exhibited a trend toward higher leak rates than non-rectal ones; other surgical and tumor-related parameters were comparable. In multivariable Cox regression analysis, AL was independently associated with decreased OS (HR 2.97; 95% CI 1.11–7.93; *p* = 0.030). After adjusting for age, UICC stage, R-status, emergency presentation, diabetes, and immune status, Kaplan–Meier curves confirmed shorter survival in patients experiencing AL.

**Conclusion:**

AL is an independent factor negatively influencing OS of colon cancer. These findings highlight the value of perioperative strategies reducing AL incidence.

## Introduction

Colorectal cancer (CRC) is the third most common cancer worldwide and contributes significantly to global cancer mortality [1]. Management strategies differ between colon and rectal cancer due to variations in prognosis and treatment approaches. Quality and extent of surgical intervention play a pivotal role in determining long-term oncologic outcomes.

Postoperative complications—most notably anastomotic leakage (AL)—remain a major determinant of both short- and long-term outcomes after colorectal surgery. Clinically, AL is associated with increased postoperative morbidity, prolonged hospitalization, higher reintervention and stoma rates, and substantial health-economic burden [2]. Importantly, AL can compromise cancer care pathways: several studies and reviews report that leaks and related septic complications may delay or reduce compliance with adjuvant therapy, which is itself time-sensitive for oncologic benefit [3].

A key consideration is that AL risk is not uniform across colorectal procedures. In general, rectal resections—particularly low anterior resection with low pelvic/coloanal anastomoses—show higher AL rates than colon resections, reflecting technical complexity in the narrow pelvis, more tenuous distal perfusion, and the frequent influence of neoadjuvant radio-chemotherapy in rectal cancer [4]. This difference is also evident in contemporary cohorts reporting markedly higher leakage proportions after rectal compared with colonic resections. Consequently, when interpreting AL-related outcome data, it is critical to stratify by anatomic site and procedure type, as pooling colon and rectal resections can obscure clinically meaningful differences.

Despite the strong biological and clinical rationale linking AL to inferior oncologic outcomes (via inflammatory activation, immunomodulation, postoperative deconditioning, and treatment delays), the literature remains heterogeneous. Reported associations with overall survival, disease-free survival, and recurrence vary across studies, with some analyses demonstrating worse survival after AL and others suggesting more nuanced effects (e.g., predominantly early mortality effects or context-dependent associations) [5]. This inconsistency is amplified by differences in study design, case mix, surgical technique, leak detection intensity, and—critically—non-uniform definitions and grading of AL. Adoption of standardized criteria such as the International Study Group of Rectal Cancer (ISREC) definition and severity grading has improved comparability, but variability in implementation persists across datasets and eras [6].

To address this, we conducted an analysis of the Wuerzburg Tumor Registry, examining the association between anastomotic leakage and overall survival after colon cancer resection. We explicitly excluded patients with rectal cancer. By providing additional data from a single-center cohort, this study aims to enhance understanding of AL’s impact on prognosis and to develop strategies for optimizing patient outcomes after colonic cancer resection.

## Methods

All patients who underwent surgical resection for colon cancer between January 1, 2019, and December 31, 2021, were identified using the institutional tumor documentation system (Onkostar) of the University Hospital of Wuerzburg (UKW). Within this system, all patients diagnosed with a malignant disease coded according to ICD-10 (C diagnoses) are prospectively and comprehensively recorded as part of routine oncologic documentation. Data collection was initially conducted within the framework of the P-Study and subsequently used for the present analysis. The P-Study was initiated through the GZO Hospital Wetzikon as a retrospective data collection effort involving national and international surgical centers. The full working title is “Development of machine learning models for the prediction of complications after colonic, colorectal, and small intestine anastomosis in psychiatric and non-psychiatric patient populations.” An ethics approval from the Ethics Committee of the Canton of Zurich is available under BASEC No. 2021–02105.

Colon carcinomas were identified based on ICD-10 coding for the purpose of this study. Patients with C20 (rectal cancer) and patients with appendiceal carcinoma who underwent appendectomy alone were explicitly excluded.

The University Hospital of Wuerzburg as a tertiary referral hospital is a certified Comprehensive Cancer Center (CCC Mainfranken) and an accredited Visceral Oncology Center, with continuous certification as a colorectal cancer center since 2008. All patients included in the present analysis were treated within these certified structures, ensuring standardized diagnostic pathways, interdisciplinary tumor board discussion, and guideline-conform oncologic management.

Clinical, surgical, and oncologic data were initially extracted from the tumor documentation system and transferred into structured Excel spreadsheets. Where necessary, missing or ambiguous information was supplemented by review of the hospital information systems, including electronic medical records, operative reports, discharge summaries, and pathology reports. Data collection and validation were completed by the end of 2023. Follow-up data were updated until June 30, 2025, which constituted the date of last follow-up for survival and outcome analyses.

During the evaluation period, a high standard of surgical quality was maintained within our certified colorectal cancer center and was regularly monitored through audits conducted by the German Cancer Society. In accordance with current guidelines, total mesocolic excision (CME) with central vascular ligation and systematic D3-lymphadenectomy was performed as part of the surgical procedure, and the number of examined lymph nodes met the guideline-recommended threshold of more than 12. Vascular ligation was performed using a low-tie technique. The decision between an open or minimally invasive approach was based on individual patient characteristics, including comorbidities, body mass index, and the extent of tumor involvement suspected on preoperative diagnostic imaging. Based on the prevailing evidence at the time, right-sided hemicolectomy was routinely performed via an open approach, as multicenter analyses had indicated a reduced lymph node yield associated with laparoscopic resection[7]. In addition to elective procedures, emergency operations—such as those performed for large bowel obstruction or perforated tumors—were also included to better reflect clinical reality. Based on current literature, all elective patients received preoperative antibiotic decontamination. Mechanical bowel preparation was not performed prior to colon resections [8, 9]. Intraoperatively, all anastomoses are assessed for integrity, absence of tension, and adequate perfusion. In addition, rectal anastomoses are further evaluated using an air leak test and a povidone-iodine leak test.

Anastomotic leakage (AL) was defined as a postoperative complication requiring therapeutic intervention—either surgical reoperation, endoscopic treatment, or intravenous therapy—and accompanied by a clinically relevant deterioration in the patient’s general condition. This definition focuses on clinically significant leaks with tangible consequences for patient management and outcome [6, 10]. The diagnosis of AL was established by means of computed tomography or endoscopy, depending on the location of the anastomosis. In patients who underwent surgery due to significant clinical deterioration, the presence of AL was additionally confirmed intraoperatively. Patients presenting with isolated intra-abdominal fluid collections without the presence of air adjacent to the anastomosis were not classified as having an AL. AL classified as Grade A according to the International Study Group of Rectal Cancer classification was not included in the analysis.

For analytical clarity, anastomoses were categorized into two groups based on their anatomical location: rectal anastomoses and non-rectal (colonic) anastomoses. This distinction was chosen a priori, as previous studies have consistently demonstrated higher anastomotic leakage rates following rectal anastomoses compared with colon or ileal anastomoses, attributable to anatomical, technical, and treatment-related factors. Stratification by anastomotic location was therefore considered essential to account for procedure-specific risk profiles and to avoid confounding when analyzing postoperative outcomes. A rectal anastomosis is required particularly in patients with sigmoid carcinoma or in those with a familial genetic predisposition and a resulting indication for colectomy. This does not imply that rectal carcinomas were included.

In addition to classifying anastomoses as rectal or non-rectal, we further analyzed whether the anastomosis was constructed using a hand-sewn or stapled technique and assessed its configuration. Furthermore, patients undergoing surgery for colon carcinoma with the creation of a diverting stoma or an end colostomy were also included in the analysis, as this reflects clinical practice. A protective ileostomy was constructed at the surgeon’s discretion for intraoperative indications, including rectal anastomosis and emergency procedures. In selected cases, a primary end stoma was created, for example, in patients with pre-existing incontinence or when colectomy was indicated due to hereditary cancer predisposition. Patients without an anastomosis were assigned to the no AL group.

### Statistical analysis

All analyses were conducted using IBM SPSS Statistics (version 29, IBM Corp., Armonk, NY, USA). Overall survival (OS) was defined as interval (in months) from the date of initial diagnosis to the date of death or last follow-up. Patients who were alive at the end of the observation period or lost to follow-up were censored at their last known survival date.

Categorical variables were compared between the two study groups using Pearson’s Chi-square test. When assumptions for the Chi-square test were not met (i.e., expected cell count < 5), Fisher’s exact test was applied. All tests were two-sided, and a *p*-value < 0.05 was regarded as statistically significant.

Mean survival time with 95% confidence intervals was estimated using the Kaplan–Meier method; median survival could not be determined because fewer than 50% of events occurred during follow-up. Survival was compared between AL groups with the log-rank test. Follow-up duration was defined as the time from surgery to the last known contact or death. The median follow-up time was calculated using the reverse Kaplan–Meier method, with inversion of the censoring indicator relative to the overall survival analysis, to ensure an accurate estimation of follow-up in the presence of censored observations.

Factors influencing survival were investigated using a multivariable Cox proportional hazards regression. Time-to-event was the outcome, with censoring applied at the end of follow-up or death unrelated to the event of interest. Candidate predictors included age, tumor stage, surgical margin status, KRAS mutation status, emergency presentation, diabetes, and immunosuppressive therapy. Categorical variables were incorporated using indicator coding. A stepwise forward selection procedure based on the likelihood-ratio test was applied, with thresholds of *p* < 0.05 for entry and *p* > 0.10 for removal. Hazard ratios (HR) with 95% confidence intervals (CI) were reported, and survival curves were plotted to visualize differences between groups.

## Results

A total of 166 patients who underwent surgical resection for colon cancer were included in the analysis. Regarding sex distribution, 104 patients (62.7%) were male. The mean age at diagnosis was 67.31 years in the overall cohort. Tumor localization according to ICD-10 classification did not differ significantly between the AL and non-AL groups (*p* = 0.470). Similarly, pathological tumor stage according to the UICC classification showed no significant differences (*p* = 0.096). Most patients were diagnosed with early to intermediate disease stages, predominantly UICC stage I and IIA. Patient characteristics and oncological characteristics are shown in Tables 1 and 2.

**Table 1:** Patients characteristics of the cohort

	Patients, No (%)			*p* value
Characteristic	Total (*n* = 166)	AL (*n* = 15)	No AL (*n* = 151)	
Sex ratio, No. (M:F)	104:62	11.4	93:58	0.418
Age at diagnosis, mean (SD), y	67.31 (12.5)	67.73(13.8)	67.27 (12.4)	0.892
Diabetes	31 (18.7)	2 (13.3)	29 (19.2)	0.740
second primary cancer	40 (24.1)	5 (33.3)	35 (23.2)	0.359

Values are *n* (%) unless otherwise indicated. *SD* standard deviation, *AL* anastomotic leakage, *M:F* male to female

**Table 2:** Tumor characteristics

	Patients, No (%)			*p* value
Characteristic	Total (*n* = 166)	AL (*n* = 15)	No AL (*n* = 151)	
ICD10				
C18.0 (Cecum) C18.2 (Ascending colon) C18.3 (Flexura coli dextra) C18.4 (Transverse colon) C18.5 (Flexura coli sinistra) C18.6 (Descending colon) C18.7 (Sigmoid colon)	32 (19.3) 37 (22.3) 13 (7.8) 13 (7.8) 7 (4.2) 7 (4.2) 57 (34.3)	4 (26.7) 3 (20.0) 0 1 (6.7) 1 (6.7) 2 (13.3) 4 (26.7)	28 (18.5) 34 (22.5) 13 (8.6) 12 (7.9) 6 (4.0) 5 (3.3) 53 (35.1)	0.470
UICC				
I IIA IIB IIC IIIA IIIB IIIC	43 (25.9) 65 (39.2) 20 (12.0) 7 (4.2) 4 (2.4) 14 (8.4) 19 (11.4)	8 (53.3) 3 (20.0) 0 0 0 2 (13.3) 2 (13.3)	35 (23.2) 62 (41.1) 20 (13.2) 7 (4.6) 4 (2.6) 14 (9.3) 9 (6.0)	0.096
Grading				
G1 G2 G3 G2-3 G4	16 (9.6) 122 (73.5) 6 (3.6) 21 (12.7) 1 (0.6)	3 (20.0) 11 (73.3) 0 1 (6.7) 0	13 (8.6) 111 (73.5) 6 (4.0) 20 (13.2) 1 (0.7)	0.558
KRAS mutation	6 (3.6)	0	6 (4.0)	0.432
MSI	31 (18.7)	3 (20.0)	28 (18.5)	0.775

Values are *n* (%) unless otherwise indicated. *AL* anastomotic leakage, *ICD-10* International Classification of Diseases, 10th Revision, *UICC* according to the Union for International Cancer Control, *MSI* microsatellite instability, *KRAS* Kirsten rat sarcoma viral oncogene homolog

Complete oncologic resection with negative margins (R0) was achieved in 163 patients (98.2%) in the overall cohort. All patients in the AL group underwent R0 resection (100%), compared with 148 patients (98.0%) in the non-AL group (*p* = 0.825). The distribution of surgical approaches was comparable between patients with and without anastomotic leakage. In the AL group, 3.3% of patients underwent MIS, 60.0% open surgery, and 6.7% emergency surgery, compared with 32.5%, 61.6%, and 10.6%, respectively, in the non-AL group. No statistically significant differences were observed between groups (*p* = 0.925/0.632).

Regarding anastomotic location, 68 patients (40.0%) had a rectal anastomosis, whereas 102 patients (60.0%) had a non-rectal (colonic) anastomosis. Rectal anastomoses were more frequent in the AL group (57.9%) compared with patients without AL (37.7%); however, this difference was not statistically significant (*p* = 0.91). Perioperative characteristics are shown in Table 3.

**Table 3:** Perioperative characteristics

	Patients, No (%)			*p* value
Characteristic	Total (*n* = 166)	AL (*n* = 15)	No AL (*n* = 151)	
**Surgery**				
MIC Open Conversion Emergency	54 (32.5) 102 (61.4) 9 (5.4) 17 (10.2)	5 (33.3) 9 (60.0) 1 (6.7) 1 (6.7)	49 (32.5) 93 (61.6) 9 (6.0) 16 (10.6)	0.925 0.632
R0	163 (98.2)	15 (100)	148 (98.0)	0.859
**Anastomosis**				
Rectal Not rectal	67 (40.4) 99 (59.6)	7 (46.7) 8 (53.3)	60 (39.7) 91 (60.3)	0.602
Hand sewn Stapled	17 (11.2) 135 (88.8)	2 (13.3) 13 (86.7)	15 (10.9) 122 (89.1)	0.781
End to end Side to end Side to side End to side	57 (37.5) 38 (25.0) 56 (36.8) 1 (0.7)	6 (40.0) 3 (20.0) 6 (40.0) 0	51 (37.2) 35 (25.5) 50 (36.5) 1 (0.7)	0.863
Stoma Protective ileostomy Primary end-stoma Double-barrel stoma	30 (18.1) 16 11 3	1 (6.7) 1 0 0	29 (19.2) 15 11 3	0.229
Ileus	37 (22.3)	5 (33.3)	32 (21.2)	0.288
LOS (SD) days	14.9	29.6 (21.9)	13.6 (8.7)	0.013

Values are *n* (%) unless otherwise indicated. *SD* standard deviation, *AL* anastomotic leakage, *MIC* minimally invasive surgery, *LOS* length of stay

Among the 15 patients with AL, adjuvant chemotherapy was recommended by the interdisciplinary tumor board for three patients. Timely initiation of chemotherapy was documented in one patient. One patient declined chemotherapy, and one patient did not receive chemotherapy due to a complicated clinical course. In the AL group, there were no deaths within 30 days and one death within 90 days as shown in Table 4.

**Table 4:** Patients with Anastomotic leakage

Nr	Tumor location	UICC	Anastomosis technique	Anastomosis direction	Day of AL	Therapy of AL (ISREC Grade)	30 d mortality	90 d mortality
1	Cecum	I	Stapled	Side to side	4	Reoperation (C)	No	No
2	Cecum	IIIC	Stapled	Side to side	8	Reoperation (C)	No	No
3	Cecum	I	Hand-sewn	Side to end	19	Antibiotics (B)	No	No
4	Cecum	IIIB	Stapled	Side to side	9	Reoperation (C)	No	No
5	Ascending colon	IIIC	Stapled	Side to side	8	Reoperation (C)	No	No
6	Ascending colon	I	Stapled	Side to side	12	Reoperation (C)	No	No
7	Ascending colon	IIA	Stapled	Side to side	11	Reoperation (C)	No	No
8	Transverse colon	IIA	Stapled	End to end	8	Reoperation (C)	No	No
9	Flexura coli sinistra	I	Hand-sewn	End to end	9	Reoperation (C)	No	No
10	Descending colon	I	Stapled	End to end	5	Reoperation (C)	No	No
11	Descending colon	I	Stapled	End to end	5	Antibiotics (B)	No	No
12	Sigmoid colon	IIA	Stapled	Side to end	10	Antibiotics + rectal drainage (B)	No	Yes
13	Sigmoid colon	I	Stapled	Side to end	6	Reoperation (C)	No	No
14	Sigmoid colon	I	Stapled	End to end	8	Reoperation (C)	No	No
15	Sigmoid colon	IIIB	Stapled	End to end	3	Reoperation (C)	No	No

*UICC* according to the Union for International Cancer Control, *AL* anastomotic leakage

**Fig. 1:**
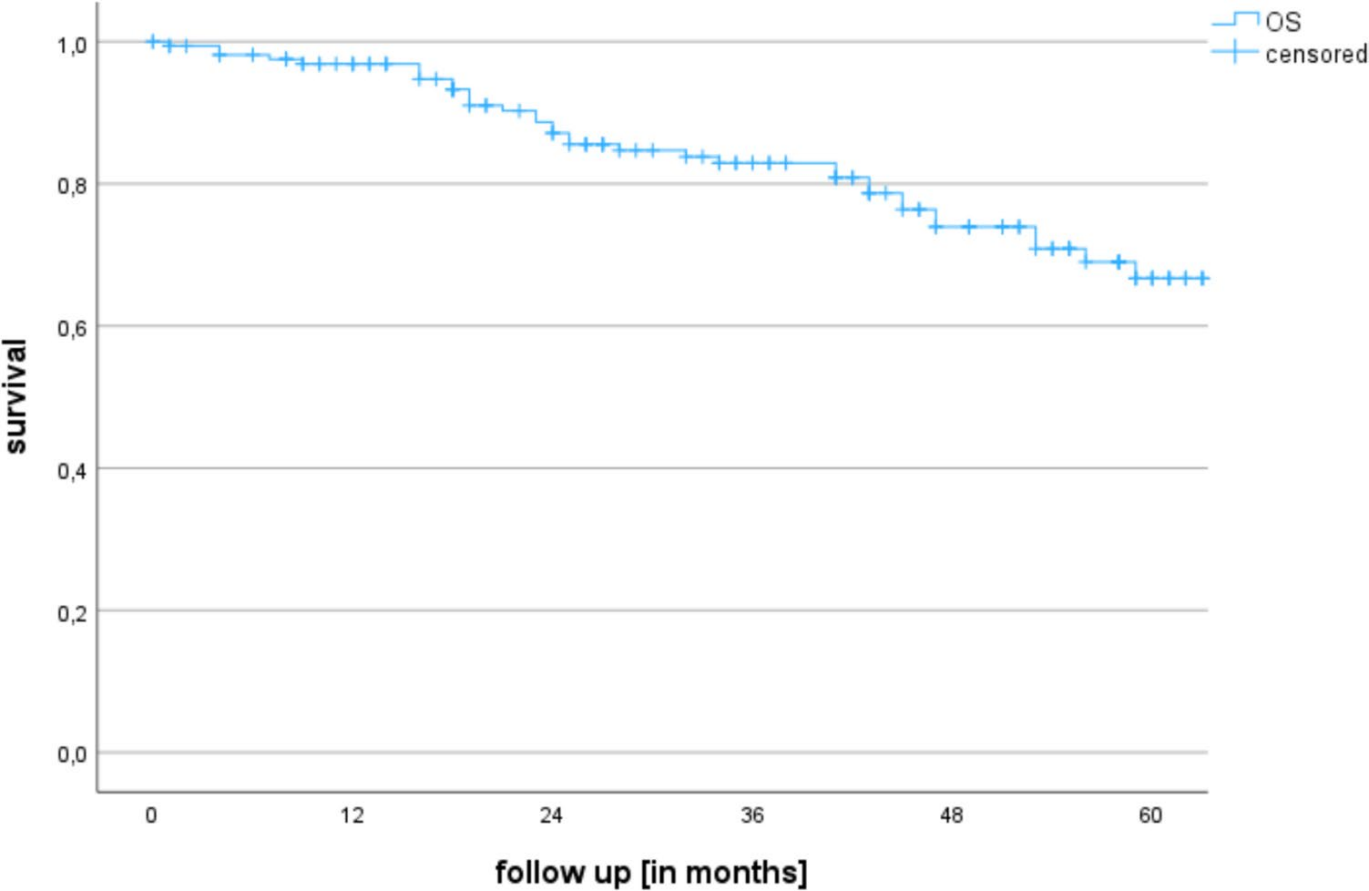
Kaplan–Meier curve of overall survival of all patients undergoing surgery

**Fig. 2:**
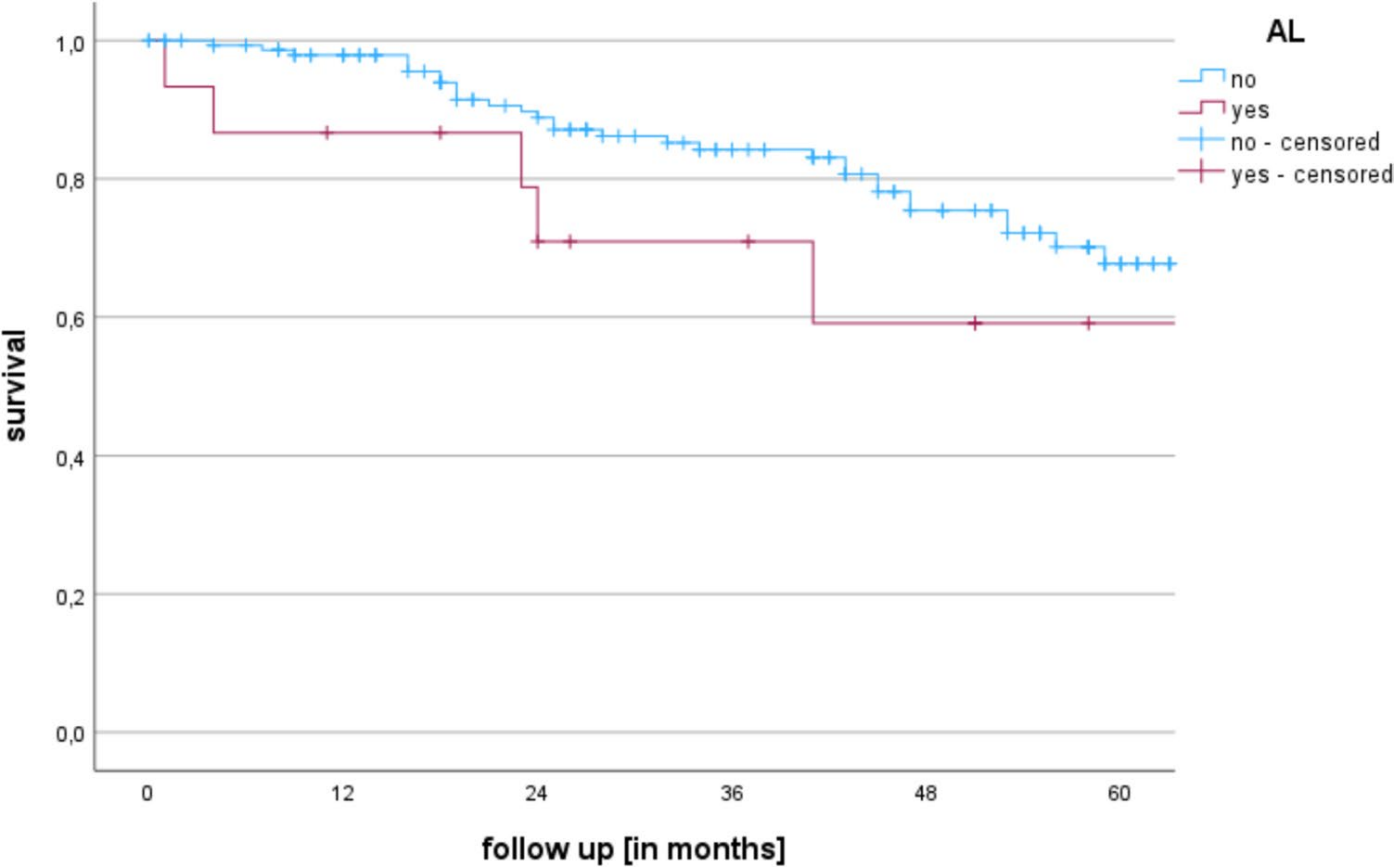
Kaplan–Meier curve of survival stratified by the occurrence of an anastomotic leakage

The mean overall survival (OS) of the entire cohort was 59.7 months (95% CI 55.6–63.7). The mean overall survival was 60.5 months (95% CI 56.4–64.6) in patients without AL and 47.8 months (95% CI 34.9–60.7) in those with AL (Figs. 1 and 2, Table 5).

**Table 5:** Survival

	Patients, No (%)			*P* value
Characteristic	Total (*n* = 166)	AL (*n* = 15)	No AL (*n* = 151)	
OS (month) Mean (95%CI) Median (95%CI)	59.7 (55.6–63.7) -	47.8 (34.9–60.7) -	60.5 (56.4–64.6) -	0.150
Follow up time (months) Mean (95%CI) Median (95%CI)	41.6 (38.4–44.9) 46 (40.8–51.2)	41.9 (31.5–52.3) 51 (39.9–62.0)	41.6 (38.2–45.0) 46 (40.8–51.2)	0.767

Values are *n* (%) unless otherwise indicated. *95%CI* 95% confidence interval, *AL* anastomotic leakage, *OS* overall survival

Univariable Kaplan–Meier survival analysis stratified by AL status demonstrated no statistically significant difference in overall survival between patients with and without AL (log-rank *p* = 0.150) (Fig. 2).

Cox proportional hazards regression was performed to evaluate the association of clinical factors with overall survival. In the final multivariable Cox regression analysis (Table 6), AI (HR 2.97, 95% CI 1.11–7.93, *p* = 0.030), age (HR 1.05 per year, 95% CI 1.02–1.08, *p *= 0.001), R-status (HR 2.83, 95% CI 1.07–7.45, *p* = 0.035), and emergency surgery (HR 3.61, 95% CI 1.59–8.16, *p* = 0.002) were independently associated with reduced overall survival.

**Table 6:** Multivariable Cox proportional hazards regression analysis identifying independent predictors of overall survival (final model)

Variable	HR (Exp(B))	95% CI for HR	Wald χ^2^	*p*-value
AL (anastomotic leakage)	2.97	1.11–7.93	4.687	0.030
Age (per year)	1.05	1.02–1.08	10.120	< 0.001
R (resection status)	2.83	1.07–7.45	4.425	0.035
Emergency surgery	3.61	1.59–8.16	9.465	0.002

Hazard ratios (HR) with 95% confidence intervals (CI), Wald chi-square statistics, and p-values are reported. Variables were selected using backward stepwise likelihood ratio method

**Fig. 3:**
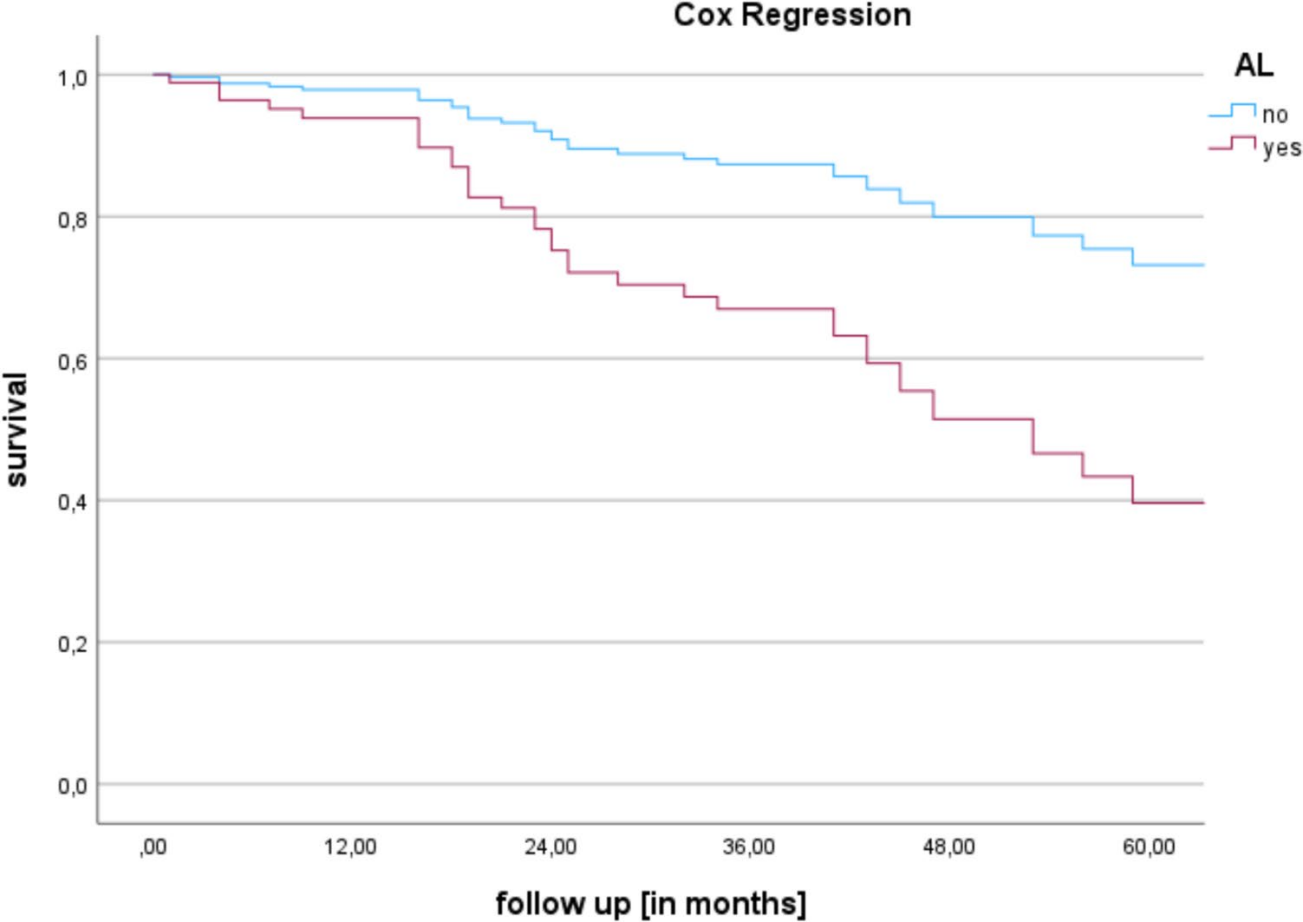
Cox proportional hazards regression analysis of the effect of anastomotic leakage on overall survival

The Kaplan–Meier curve derived from the Cox regression analysis showing the impact of AL is presented in Fig. 3, while the variables included in the regression model are summarized in Table 7.

**Table 7:** Multivariable Cox proportional hazards regression analysis identifying independent predictors of overall survival (full model)

Variable	HR (Exp(B))	95% CI for HR	Wald χ^2^	*p*-value
AL (anastomotic leakage)	2.85	1.05–7.69	4.527	0.039
Age (per year)	1.06	1.02–1.09	11.178	< 0.001
R (resection status)	3.35	1.05–10.68	4.166	0.041
Emergency surgery	2.86	1.21–6.75	5.759	0.016
Diabetes mellitus	2.04	0.75–5.77	1.814	0.178
UICC stage	1.12	0.94–1.34	1.646	0.200
Immunosuppression	1.42	0.38–5.25	0.270	0.603

Hazard ratios (HR) with 95% confidence intervals (CI), Wald chi-square statistics, and *p*-values are reported. Variables were selected using backward stepwise likelihood ratio method

Additionally, patient-specific risk factors potentially affecting the occurrence of anastomotic leakage were assessed. Using a Cox proportional hazards model with backward stepwise likelihood-ratio selection, rectal anastomosis was associated with an increased risk of anastomotic leakage, although this effect was not statistically significant (*p* = 0.98). The other factors examined (gender, age, UICC, type of surgery, diabetes, immune status, emergency surgery, or hand-sewn vs. stapled anastomosis) did not provide any indication of a negative impact.

## Discussion

In our cohort of 166 patients undergoing colon resection, anastomotic leakage was observed in 15 patients. Importantly, patients who developed an anastomotic leak experienced a clinically relevant reduction in overall survival, with a median survival decrease of 12.7 months. Among various potential predictors—including age, tumor stage, resection status, emergency presentation, diabetes, and immune status—AL remained a significant factor in the multivariable Cox regression analysis. This finding highlights the particularly detrimental influence of AL on prognosis. Consequently, strategies to reduce AL are crucial to improve outcome in colon cancer patients.

An important consideration when interpreting the association between anastomotic leakage and reduced overall survival is the potential confounding effect of patient-related factors. Patients with impaired baseline health status may be inherently more susceptible to postoperative complications and, independently, have a shorter life expectancy. Several studies have demonstrated that comorbidities such as diabetes, cardiovascular disease, pulmonary disease, malnutrition, or immunosuppression can compromise wound healing and increase the risk of AL. Consequently, the observed association between AL and reduced survival may in part reflect underlying patient vulnerability rather than a direct causal oncologic effect of the leakage itself. In our cohort, comorbidities were slightly more frequent among patients who developed AL; however, these differences did not reach statistical significance in multivariable analyses. Nevertheless, this observation underscores the importance of careful preoperative risk stratification and optimization, as patients at higher baseline risk may be disproportionately affected by both AL and reduced long-term survival [11].

Beyond patient-related factors, AL remains one of the most severe complications following colorectal surgery and is consistently associated with increased postoperative morbidity, prolonged hospital stay, reinterventions, and early mortality [12, 13]. In recent years, AL has also been increasingly recognized as a potential determinant of long-term oncologic outcomes. Several mechanisms have been proposed to explain this association, including sustained systemic inflammatory responses, impaired immune surveillance, delayed or omitted adjuvant therapy, and prolonged postoperative deconditioning. However, the magnitude and nature of the long-term impact of AL remain controversial, particularly in colon cancer. Importantly, the relevance of delayed adjuvant therapy as a mediating factor differs substantially between colon and rectal cancer.

In rectal cancer, most patients currently receive total neoadjuvant therapy (TNT), integrating systemic chemotherapy and radiotherapy prior to surgery. As a consequence, postoperative adjuvant therapy is frequently not required, and treatment completion is achieved before surgical resection. In this setting, anastomotic leakage does not typically result in delayed or omitted systemic therapy and therefore cannot be considered a major driver of impaired overall survival. In contrast, adjuvant chemotherapy remains a cornerstone of treatment in colon cancer, particularly in stage III disease, and postoperative complications such as AL may directly interfere with the timely initiation or completion of adjuvant therapy. This fundamental difference further supports the need to interpret the oncologic impact of AL separately for colon and rectal cancer and underscores the relevance of colon-specific analyses when evaluating survival outcomes.

A major source of heterogeneity in the existing literature is the frequent pooling of colon and rectal cancer resections, despite well-established anatomical, technical, and oncologic differences between these procedures. Rectal resections, especially low anterior resections, are associated with higher AL rates and distinct recurrence patterns, largely due to pelvic anatomy, compromised perfusion, and the frequent use of neoadjuvant therapy. Recent high-quality data support this distinction: population-based analyses and randomized trial data have demonstrated that the negative oncologic impact of AL is considerably more pronounced after rectal cancer surgery, whereas the effect after colon cancer resection appears weaker or inconsistent [14–18]. Failure to stratify by tumor location may therefore obscure clinically relevant differences and substantially contribute to inconsistent findings across studies.

To avoid this important source of bias, our study explicitly focused on colon cancer resections only. By excluding rectal cancer, we minimized confounding related to technically more demanding pelvic anastomoses and ensured that the prognostic impact of AL was evaluated within a homogeneous surgical population. This approach represents a key methodological strength of the present analysis and allows for a more precise interpretation of leakage-related outcomes in colon cancer specifically.

A Spanish working group analyzing patient data from 2010 to 2019 reported a significant deterioration in overall survival associated with AL [11]. Similarly, a large Scandinavian cohort study including more than 22,000 patients from 2008 to 2012 found that AL negatively influenced survival in patients with stage III disease, while no significant effect was observed in stages I and II [19]. A systematic review published in 2020 summarizing eight studies also demonstrated a negative impact of AL on survival following colon resection [20]. Furthermore, a registry-based study from Lübeck analyzing data from 11,222 patients using propensity score methods confirmed the detrimental effect of AL on overall survival [21]. Taken together, these studies support our findings that AL represents a major determinant of long-term outcomes after colorectal surgery, even though most of these analyses were based on mixed cohorts of colon and rectal cancer, whereas our data reflect this effect specifically in colon cancer alone.

Despite this consistency, important limitations of the existing evidence must be acknowledged. Several frequently cited colon-specific analyses, including those by Marra et al. [22] and Kube et al. [23], were published more than a decade ago and therefore reflect surgical techniques, perioperative management strategies, and oncologic treatment standards that differ substantially from contemporary practice. Advances in minimally invasive surgery, enhanced recovery pathways, perioperative care, and systemic therapy may have altered the long-term consequences of AL. More recent data, such as the study by da Silva et al. [20], provide an updated perspective; however, this investigation did not demonstrate a statistically significant long-term survival disadvantage for patients with AL once early postoperative mortality was excluded. This finding suggests that the prognostic impact of AL may be attenuated in modern cohorts and highlights the need for contemporary, colon-specific data to better define its true long-term effect.

A recent study published in 2025, which included both colon and rectal resections, reported significantly increased 30-day mortality among patients with AL [24]. In contrast, early postoperative mortality did not differ between patients with and without AL in our cohort, and there was only one death within 30 days in the entire cohort, which occurred in the non-AL group. Early mortality therefore cannot explain the observed differences in overall survival in our analysis, supporting the hypothesis that longer-term systemic effects, postoperative deconditioning, or disruptions in oncologic care pathways contribute to the reduced survival observed after AL.

The incidence of AL in our cohort (9%) is within the range reported in the literature, which varies between 3 and 15% depending on patient characteristics, surgical technique, and the definition of leakage [12]. Baseline demographic and tumor-related variables were largely comparable between patients with and without AL. A higher rate of postoperative ileus was observed in patients with AL, which is most plausibly interpreted as a consequence of leakage rather than a predisposing factor.

In our cohort, UICC stage was not identified as an independent predictor of overall survival, in contrast to anastomotic leakage, age, resection status, and emergency surgery. Although this appears to differ from the established literature, it can likely be explained by the specific characteristics of our study population, which was limited to stage I–III colon cancer. By excluding patients with metastatic disease, the overall prognostic separation between stages is reduced. In addition, the relatively small number of events and the strong impact of perioperative factors, such as anastomotic leakage and emergency procedures, may have outweighed the influence of tumor stage in the multivariable model. Differences in adjuvant treatment across stages may also have contributed to a partial alignment of outcomes. Therefore, while UICC stage remains a well-established prognostic factor, its effect may be less pronounced in selected non-metastatic cohorts such as ours.

This study has limitations inherent to its retrospective, single-center design, including potential selection bias and limited generalizability. In addition, the relatively small number of anastomotic leakage (AL) events may affect the stability of the multivariable Cox regression model and limit the ability to detect weaker associations with other prognostic factors. These aspects should be considered when interpreting the results. Despite these limitations, the strengths of this study include comprehensive follow-up, high-quality tumor registry data, and the use of both forward and backward stepwise Cox regression analyses, allowing for a robust assessment of the prognostic impact of AL in a contemporary, colon cancer-specific cohort.

In summary, anastomotic leakage may be a major determinant of overall survival after colon cancer resection. Our colon-specific data confirm this association without confounding from rectal cancer, emphasizing the importance of high-quality surgery for optimal oncologic outcomes.

## Data Availability

The data that support the findings of this study are derived from the institutional tumor registry (Onkostar) and hospital information systems of the University Hospital of Würzburg. Due to patient confidentiality and legal restrictions, these data are not publicly available. Access may be granted upon reasonable request and with permission from the University Hospital of Würzburg.
